# Linking psychological capital to job embeddedness among nurses: evidence from Egyptian public healthcare setting

**DOI:** 10.1186/s12912-025-03547-2

**Published:** 2025-07-15

**Authors:** Ayman Mohamed El-Ashry, Mahmoud Abdelwahab Khedr, Mona Metwally El-Sayed, Islam Sameh Abdelhay, Mennat-Allah G. Abou Zeid, Bashair Mohamed Elsayed Abdo

**Affiliations:** 1 Psychiatric and Mental Health Nursing Department, Faculty of Nursing, Alexandria University, Alexandria, Egypt; 2 Nursing Administration Department, Faculty of Nursing, Mansoura University, Dakahlia, Egypt; 3 Nursing Administration Department, Faculty of Nursing, Ain Shams University, Cairo, Egypt; 4 Nursing Administration Department, Faculty of Nursing, Damanhour University, Beheira, Egypt; 5 College of Nursing, Prince Sattam Bin Abdulaziz University, Al-Kharj, Saudi Arabia

**Keywords:** Psychological capital, Job embedding, Nurses

## Abstract

**Background:**

Psychological capital, encompassing hope, self-efficacy, resilience, and optimism, is increasingly recognized as a critical resource in enhancing workplace engagement and retention. In high-stress professions such as nursing, psychological capital may serve as a buffer against job demands. However, limited research exists in Egypt exploring how psychological capital influences job embeddedness, a construct reflecting an employee’s attachment to their job and organization.

**Objective:**

This study aimed to examine the relationship between psychological capital and job embeddedness among nurses working in governmental hospitals in Egypt.

**Design:**

A cross-sectional, descriptive correlational design was employed, adhering to the STROBE guidelines.

**Methods:**

Data were collected from 431 registered nurses working across two public hospitals over three months. Standardized tools were used, including the 24-item Psychological Capital Questionnaire and the Global Job Embeddedness Scale.

**Results:**

Psychological capital showed a significant positive correlation with job embeddedness (*r* = 0.356, *p* < 0.001). Regression analysis confirmed PsyCap as a significant predictor of JE (*B* = 0.317, *p* < 0.001), accounting for 19.8% of the variance (*R²* = 0.198). Among PsyCap dimensions, optimism had the strongest individual correlation with JE (*r* = 0.406, *p* < 0.001). Conversely, patient load per shift was negatively associated with JE (*B* = − 0.269, *p* < 0.001).

**Conclusion:**

Psychological capital is a significant linked to job embeddedness among Egyptian nurses. Nurses with higher psychological capital levels are more likely to feel anchored in their roles, whereas high patient loads erode this attachment.

**Nursing implications:**

Healthcare institutions should implement programs to develop nurses’ psychological capital particularly resilience, optimism, and self-efficacy. In parallel, managing nurse workloads is essential to enhancing retention, improving work satisfaction, and ensuring sustained quality care in challenging healthcare environments.

**Clinical trial number:**

Not applicable.

## Introduction

Psychological Capital (PsyCap) has become a central construct within positive organizational behavior, highlighting employees’ beneficial psychological resources in the workplace. Luthans et al. (2007) conceptualized PsyCap as an individual’s “positive psychological state of development,” encompassing four dimensions: hope, efficacy (self-efficacy), resilience, and optimism—collectively known as the HERO framework [1]. These elements are characterized by their malleable and state-like nature, meaning they can be enhanced over time through targeted organizational interventions. Specifically, hope refers to persistently striving toward goals and adjusting pathways when required; efficacy represents individuals’ belief in their capability to meet challenges; resilience is the ability to effectively recover from setbacks; and optimism denotes a positive expectation of achieving success both now and in the future [1]. Together, these PsyCap resources equip employees to effectively handle workplace demands and proactively strive toward desired outcomes. Unlike fixed personality traits, PsyCap can be developed through organizational support and targeted training initiatives [1, 2]. Thus, PsyCap has gained prominence in studies focused on improving employees’ performance, satisfaction, and well-being, especially within high-pressure professions such as nursing.

A substantial body of evidence demonstrates the critical role of PsyCap in improving employees’ attitudes, behaviors, and performance. Employees with higher PsyCap exhibit greater job satisfaction, work engagement, and organizational commitment while experiencing lower levels of stress and turnover intent [3]. Within nursing, higher PsyCap is particularly beneficial, as it is associated with lower burnout, reduced stress levels, and increased career satisfaction, ultimately supporting better retention and enhanced patient care [4].

As PsyCap has gained increasing attention, exploring its impact on employee retention has become vital [1]. The concept of job embeddedness (JE) provides a useful theoretical lens for understanding retention by highlighting the various forces that anchor employees to their organizations. Mitchell et al. (2001) describe JE through three interconnected dimensions: fit (alignment with organizational and community values), links (interpersonal relationships at work and in the community), and sacrifice (costs associated with leaving the job) [5]. Unlike traditional predictors such as job satisfaction or turnover intention, JE encompasses a broader scope of factors, providing a comprehensive understanding of retention dynamics.

Within nursing, JE has been effectively utilized to address persistent turnover issues by emphasizing factors that motivate nurses to remain, rather than focusing exclusively on reasons for leaving [6]. Nurses develop professional alignment (fit), strong interpersonal relationships with colleagues and patients (links), and tangible benefits (sacrifice), which increase their job embeddedness. Prior research highlights the need to further explore the antecedents of nurses’ embeddedness, noting that supportive leadership and personal resources, such as self-efficacy, enhance nurses’ retention [6].

The interrelationship between Psychological Capital (PsyCap) and Job Embeddedness (JE) among nurses is a critical area of study with significant implications for healthcare organizations. PsyCap, which encompasses optimism, hope, self-efficacy, and resilience, appears to play a crucial role in enhancing nurses’ JE. Optimistic and hopeful nurses are more likely to envision a positive future in their workplace, leading to a stronger sense of organizational fit. Those with high self-efficacy tend to proactively build robust professional networks and engage more actively in their roles, thereby strengthening their links within the organization. Resilient nurses, capable of bouncing back from challenges, may perceive fewer incentives to leave their positions, as they view job difficulties as less of a sacrifice [6, 7].

Empirical evidence from diverse cultural contexts, including studies conducted in China and Egypt, has consistently demonstrated a positive correlation between PsyCap and JE [8, 9]. These findings suggest that nurses with higher levels of psychological resources become more deeply embedded in their roles, leading to higher retention rates. Furthermore, recent research has revealed that PsyCap’s positive impact on nurses’ job performance is mediated by increased job embeddedness [10]. This indicates that firmly embedding nurses within their organizational roles is a crucial mechanism for achieving enhanced workplace outcomes. These insights underscore the importance of fostering PsyCap among nursing staff as a strategic approach to strengthen their connection to the organization and ultimately improve overall healthcare delivery and organizational effectiveness.

### Significance and contextual novelty of the study

Despite existing evidence supporting the PsyCap-JE relationship, most research has emerged from Western or East Asian contexts, potentially limiting its generalizability. Egyptian healthcare faces unique and substantial challenges, such as chronic staffing shortages, excessive patient loads, limited managerial support, inadequate recognition, limited career growth opportunities, and significant nurse migration [6, 11, 12]. These specific contextual factors offer a novel and compelling backdrop for exploring PsyCap’s role in enhancing nurses’ attachment to their organizations.

Moreover, Egypt’s distinct cultural setting—characterized predominantly by collectivism, where interpersonal relationships, social integration, and community ties hold considerable importance—likely influences the “links” dimension of JE differently than in more individualistic cultures [6]. The strong emphasis on social and relational connections may amplify the impact of psychological capital on nurses’ attachment, making the study of PsyCap-JE particularly relevant and novel in this cultural context.

By explicitly investigating the PsyCap-JE relationship within Egyptian public hospitals, this study addresses a critical gap, providing culturally sensitive insights into retention dynamics among nurses. Additionally, comparing these findings with of those future studies on nurses in Egyptian private hospitals or other Arab nations can further enhance the understanding of the cultural specificity and generalizability of PsyCap’s role in retention strategies. Thus, this research contributes novel, contextually rich knowledge, offering implications that extend beyond existing international studies, thereby informing targeted nurse retention policies tailored to culturally distinctive health care environments.

### Study objective

- To explore the link between psychological capital and job embeddedness among nurses.

### Researh question

- What is the relationship between psychological capital and job embeddedness among nurses?

## Methods

### Research design

A descriptive, correlational, cross-sectional survey design was used, adhering to the Strengthening the Reporting of Observational Studies in Epidemiology (STROBE) guidelines. Data collection occurred over three months from early October to late December 2024, arranged flexibly to align with participants’ availability and schedules.

### Setting

The study data was collected from a diverse group of nurses across two hospitals in El-Behiara: Damanhour General and Itay El-Baroud Hospitals. These hospitals, affiliated with the Ministry of Health and Population, were chosen for their representative nature. The study included three ICUs - general, intermediate care, and emergency care unit - and seven medical departments - general medical units (male and female), pediatric, isolation, endoscopic, hematemesis, toxicology, and dialysis units. This comprehensive approach to data collection ensures a robust and nuanced understanding of the research topic.

### Target participants: inclusion and exclusion criteria

To be eligible for the study, participants had to hold either a diploma or a bachelor’s degree in nursing and have at least one year of clinical experience. Nurses still in training or recently returning from extended leave, such as maternity leave, were excluded from the study.

### Sample size Estimation

The sample size was determined using the G*Power software (version 3.1.9.7 A) based on the following parameters: a statistical power of 0.95, an effect size of 0.25, a significance level (α) of 0.05, with one group being analyzed, and an anticipated dropout rate of approximately 20% [13]. Based on these criteria, the calculated minimum required sample size was 420 participants.

### Sampling and recruitment

The recruitment process involved selecting 451 nursing staff from various departments, including intensive care units (general, intermediate care, and emergency care) and seven medical departments (general medical units for both male and female patients, pediatric unit, isolation unit, endoscopic unit, hematemesis unit, toxicology unit, and dialysis unit). However, 20 nurses were excluded from the study due to not meeting the inclusion criteria—8 were still in training, 4 had recently returned from maternity leave, and 8 declined to participate. Consequently, the final study sample comprised 431 nursing staff, yielding a response rate of 95.5% and a dropout rate of 4.5%.

### Study instruments

Three instruments were used.

### Demographic datasheet

This sheet included questions about the nurses’ gender, age, marital status, educational level, working hours/week, monthly income, and workplace area.

### Psychological capital questionnaire (PCQ)

This study utilized the 24-item Psychological Capital Questionnaire (PCQ) developed by Luthans et al. (2017). The instrument consists of four components: optimism, resilience, hope, and self-efficacy, with six items measuring each construct. The total scores of the Psychological Capital Questionnaire (PCQ), which consists of 24 items, each rated on a 5-point Likert scale (1 = strongly disagree to 5 = strongly agree) ranges from 24 to 120. Based on the distribution, scores were categorized into three levels: Low: Mean score < 2.50, moderate: mean score from 2.50 to < 3.75, and high mean score ≥ 3.75, following tertile cutoffs to assess the degree of psychological capital. Additionally, it is essential to mention that Mind Garden permitted the researchers in this study to use the PCQ (from https://www.mindgarden.com). The scale showed good internal consistency in the study of Da et al. (2021) with Cronbach’s α of 0.82 [14]. In this study, the reliability estimate using Cronbach’s alpha was 0.78.

### Global job embeddedness scale (GJES)

The Global Job Embeddedness Scale, created by Crossley et al. (2007), comprises seven items that collectively measure the overall level of job embeddedness among nurses. Each item reflects a single-dimensional aspect of embeddedness—for instance, one item reads, “I feel attached to this hospital.” Notably, item 6 is reverse-scored. Higher total scores on the scale indicate a greater degree of job embeddedness. In the current study, the scale demonstrated strong internal consistency, with a Cronbach’s alpha of 0.86, closely aligning with the original reliability coefficient of 0.88 reported by the scale’s developers [15].

### Procedure

#### Ethical considerations

Formal authorization and consent to carry out the research was meticulously secured from the Research Ethics Committee (REC) of the Faculty of Nursing at Damanhour University, Egypt (IRB/DAMANHOUR/87 C/10/2023). Further approval was obtained from the hospital administrations after the study’s purpose and procedures were explained. Before participating, all nurses were thoroughly informed about the study’s aims, procedures, potential risks, and their rights, including the option to participate voluntarily and the freedom to withdraw at any time without any repercussions.

To protect participant anonymity during the face-to-face data collection, all questionnaires were completed privately and submitted anonymously in sealed envelopes. No identifying information was collected, and each participant was assigned a random code number known only to the researchers. Moreover, data collectors were not affiliated with hospital management and were trained to ensure that no pressure was applied during recruitment. Managers and supervisors were not involved in the administration or collection of data to prevent undue influence. These steps ensured that participants’ identities remained confidential throughout the research process.

### Validity of the instruments

Translating the research instruments, including the PCQ and GJES, into Arabic was executed carefully to guarantee precision and cultural significance. Bilingual experts proficient in both English and Arabic conducted the translations, ensuring the retention of linguistic and cultural subtleties. A back-translation method was utilized to verify the translations, where the Arabic texts were rendered back into English to check for linguistic alignment and address any inconsistencies. Subsequently, face validity evaluations were carried out, with panels of experts from pertinent fields assessing the translated instruments to confirm they accurately represented the intended constructs within the Arabic environment. Feedback from potential participants was also collected to ensure the items were clear, relevant, and culturally suitable.

Regarding PCQ, an exploratory factor analysis was performed after translating the scale from English to Arabic. The factor loadings initially varied between 0.512 and 0.873, but after varimax rotation, they improved to a range of 0.680 to 0.963, surpassing the 0.35 threshold. Overall, the analysis accounted for 78.606% of the total variance. For GJES, Following the translation of the scale into Arabic, exploratory factor analysis was conducted. Initially, factor loadings varied from 0.45 to 0.80 and, after applying varimax rotation, improved to a range of 0.63 to 0.91, which accounted for 72.24% of the total variance. The Kaiser-Meyer-Olkin (KMO) measure showed excellent sampling adequacy at 0.951, and Bartlett’s test of sphericity indicated high significance (*p* ≤ 0.001), thereby validating the factorability of the correlation matrix. All items were preserved.

To further support the structural validity of the adapted tools used in this study, a confirmatory factor analysis (CFA) was conducted for both the Psychological Capital Questionnaire (PCQ) and the Global Job Embeddedness Scale (GJES). CFA was performed using AMOS version 26.0 to assess how well the data fit the hypothesized factor structures based on the original instruments. For the PCQ, which includes 24 items, a second-order model was specified with four latent constructs—self-efficacy, hope, resilience, and optimism—each measured by six items. The model demonstrated a good fit to the data, with fit indices as follows: Chi-square (χ²) = 584.29, degrees of freedom (df) = 246, *p* < 0.001, Comparative Fit Index (CFI) = 0.947, Tucker-Lewis Index (TLI) = 0.936, Root Mean Square Error of Approximation (RMSEA) = 0.058 with a 90% confidence interval (CI) between 0.051 and 0.065, and Standardized Root Mean Square Residual (SRMR) = 0.046. All standardized factor loadings were significant (*p* < 0.001), ranging from 0.61 to 0.86, indicating that each item was a strong indicator of its corresponding latent construct.

Similarly, a CFA was conducted on the GJES to confirm its unidimensional structure. The results showed that the single-factor model fit the data well: χ² = 27.52, df = 12, *p* = 0.006, CFI = 0.984, TLI = 0.972, RMSEA = 0.051 (90% CI: 0.028–0.075), and SRMR = 0.029. The standardized factor loadings for the GJES items ranged from 0.63 to 0.84, indicating strong internal consistency and unidimensionality of the scale. All model fit indices met the commonly accepted thresholds for a good model fit (CFI and TLI ≥ 0.90, RMSEA ≤ 0.06–0.08, and SRMR ≤ 0.08), based on established guidelines [16]. Together, these CFA findings confirm the construct validity and structural integrity of the Arabic versions of the PCQ and the GJES. They also affirmed that the instruments were suitable for assessing psychological capital and job embeddedness among nurses within the Egyptian public healthcare context.

### Pilot study

The pilot study, which used a sample size of *n* = 50, assessed the applicability and clarity of the research instruments, the feasibility of fieldwork, and any potential barriers that could hinder the researcher’s ability to collect data. The results indicated that no modifications were needed.

### Data collection

Before initiating data collection, formal approval was obtained from the relevant authorities at each research site, ensuring compliance with institutional protocols and ethical guidelines. To respect participants’ time and work schedules, the trained research team coordinated with participants individually, arranging meetings during their designated break times to minimize disruption to their duties. Additionally, the researchers held separate meetings with each head nurse eligible to participate in the study to explain the study’s objectives, procedures, and significance. The meeting was carefully designed to be comprehensive and time-efficient, with an estimated completion time of 20 to 30 min.

A **questionnaire-based survey** method was used for data collection. The survey was administered in person by trained data collectors using printed, self-administered forms. Participants received a hard copy of the questionnaire, which included a cover letter explaining the study’s purpose, anonymity assurances, and voluntary nature of participation. Nurses completed the questionnaire in a quiet, private setting during break hours to reduce external pressure and allow thoughtful responses.

The research team took careful steps to prevent any form of coercion. Participants were explicitly informed—both verbally and in writing—that their participation was entirely voluntary, and that they could decline or withdraw at any point without any repercussions to their job status or evaluations. No managers or supervisors were involved in data collection, and no incentives were provided. These measures ensured autonomy and minimized the risk of bias due to perceived institutional pressure.

The final study sample comprised 431 nursing staff, yielding a **response rate of 95.5%** and a dropout rate of 4.5%. While this high response rate reflects the effectiveness of scheduling and engagement strategies, it may also raise concerns regarding potential response bias, which is addressed in the study limitations.

### Data analysis

Data analysis was conducted using IBM SPSS software version 26.0 and AMOS version 26.0. To summarize the demographic and study variable data, descriptive statistics such as means, standard deviations, frequencies, and percentages were utilized. Various statistical tests were applied to explore the relationships between variables. Independent samples t-tests were employed to compare two groups of normally distributed quantitative variables. At the same time, one-way analysis of variance (ANOVA) was used to assess differences among multiple categorical groups. Pearson’s correlation coefficient was calculated to evaluate the linear relationship between psychological capital (PsyCap) and job embeddedness (JE). Multiple linear regression analysis was conducted to investigate the predictive relationship between PsyCap and JE, and hierarchical regression analysis was used to assess the contribution of PsyCap in explaining the variance in job embeddedness, controlling for demographic and workload-related covariates. Statistical significance was determined at *P* < 0.05.

## Results

Table 1 presents the demographic breakdown of nurses studied, with a significant majority being female (74.2%), highlighting the gender balance in the profession. Most of these nurses fall within the 20 to under 30 age range (74.2%). Most were single (55.2%), with 52.9% having 5 to 6 family members. Educationally, 61.7% held bachelor’s degrees, and work experience was primarily less than five years (67.1%). The majority worked more than 36 h weekly (41.5%) and reported income sufficient to some extent (49.9%).

**Table 1:** Distribution of the studied sample according to demographic characteristics (*n* = 431)

Demographic characteristics	No	%
**Gender**		
Female	320	74.2
Male	111	25.8
**Age**		
< 20	4	0.9
20-<30	320	74.2
≥ 30	107	24.8
Mean ± SD	27.86 ± 6.43	
**Marital status**		
Single	238	55.2
Married	182	42.2
Divorced /Widowed	11	2.6
**Level of education**		
Technical	112	26.0
BCs	266	61.7
Master	53	12.3
**Years of experience**		
< 5	289	67.1
5-<10	84	19.5
10-<15	16	3.7
≥ 15	42	9.7
**Number of working hours /week**		
< 12	26	6.0
> 12–24	83	19.3
> 24–36	143	33.2
> 36	179	41.5
Mean ± SD	37.52 ± 15.19	
**Number of patients in your shift**		
< 4	314	72.9
≥ 4	117	27.1
Mean ± SD	4.37 ± 6.39	
**Income**		
Sufficient	66	15.3
To some extent	215	49.9
Insufficient	150	34.8
**Number of vacation last month**		
No	75	17.4
1–2	37	8.6
3–4	152	35.3
5–6	81	18.8
6–8	39	9.0
> 8	47	10.9
Mean ± SD	4.61 ± 3.86	
**Shift**		
Morning	174	40.4
Evening	10	2.3
Night	247	57.3

The analysis presented in Table 2 shows that several demographic and work-related variables were significantly associated with Job Embeddedness (JES), while fewer were related to Psychological Capital (PCQ). Specifically, marital status was significantly related to JES (F = 3.977, *p* = 0.019, η² = 0.018), with single nurses reporting higher embeddedness scores compared to married or divorced/widowed nurses. Similarly, educational level showed a significant association with JES (F = 4.447, *p* = 0.012, η² = 0.020), where nurses with technical diplomas had slightly higher embeddedness than those with higher degrees. Number of working hours per week was also significantly associated with JES (F = 3.456, *p* = 0.017, η² = 0.015), indicating that those with fewer working hours reported greater job embeddedness.

**Table 2:** Relation between study variables and demographic characteristics (*n* = 431)

Demographic characteristics	PCQ		JES		Effect Size
	M ± SD	Test of sig.	M ± SD	Test of sig.	
**Gender**					
Female	3.75 ± 0.68	t = 0.090 *p* = 0.928	3.45 ± 0.72	t = 0.339 *p* = 0.735	----------
Male	3.75 ± 0.80		3.42 ± 0.80		
**Age**					
< 20	4.52 ± 0.21	F = 2.495 *p* = 0.084	4.11 ± 0.22	F = 1.659 *p* = 0.192	-----
20-<30	3.75 ± 0.73		3.43 ± 0.74		
≥ 30	3.72 ± 0.64		3.45 ± 0.76		
**Marital status**					
Single	3.74 ± 0.76	F = 0.032 *p* = 0.969	3.53 ± 0.72	F = 3.977* *p* = 0.019*	η² = 0.018
Married	3.75 ± 0.63		3.34 ± 0.75		
Divorced /Widowed	3.78 ± 0.89		3.23 ± 0.87		
**Level of education**					
Technical	3.80 ± 0.66	F = 1.054 *p* = 0.350	3.61 ± 0.68	F = 4.447* *p* = 0.012*	η² = 0.020
BCs	3.71 ± 0.75		3.37 ± 0.75		
Master	3.83 ± 0.54		3.46 ± 0.78		
**Years of experience**					
< 5	3.75 ± 0.71	F = 1.923 *p* = 0.125	3.44 ± 0.72	F = 0.974 *p* = 0.405	-------
5-<10	3.86 ± 0.61		3.53 ± 0.75		
10-<15	3.64 ± 0.65		3.31 ± 0.96		
≥ 15	3.56 ± 0.87		3.31 ± 0.79		
**Number of working hours /weeks**					
< 12	3.83 ± 0.59	F = 2.000 *p* = 0.113	3.55 ± 0.48	F = 3.456* *p* = 0.017*	η² = 0.015
> 12–24	3.79 ± 0.73		3.61 ± 0.74		
> 24–36	3.83 ± 0.68		3.48 ± 0.77		
> 36	3.65 ± 0.73		3.32 ± 0.73		
**Number of patients in your shift**					
< 4	3.73 ± 0.68	t = 0.811 *p* = 0.418	3.52 ± 0.71	t = 3.404* *p* = 0.001*	Cohen’s d = 0.48
≥ 4	3.79 ± 0.79		3.24 ± 0.79		
**Income**					
Sufficient	4.07 ± 0.50	F = 11.365* *p* < 0.001*	3.74 ± 0.76	F = 6.571* *p* = 0.002*	η² = 0.030
To some extent	3.76 ± 0.57		3.39 ± 0.60		
Insufficient	3.59 ± 0.90		3.38 ± 0.88		
**Number of vacation last month**					
No	3.84 ± 0.85	F = 1.721 *p* = 0.128	3.61 ± 0.70	F = 2.985* *p* = 0.012*	η² = 0.013
1–2	3.80 ± 0.61		3.69 ± 0.69		
3–4	3.65 ± 0.69		3.32 ± 0.73		
5–6	3.68 ± 0.73		3.33 ± 0.80		
6–8	3.96 ± 0.37		3.56 ± 0.70		
> 8	3.79 ± 0.72		3.46 ± 0.77		
**Shift**					
Morning	3.76 ± 0.74	F = 0.086 *p* = 0.918	3.53 ± 0.71	F = 2.134 *p* = 0.120	---------
Evening	3.79 ± 0.73		3.44 ± 0.69		
Night	3.74 ± 0.68		3.38 ± 0.77		

PCQ = Psychological Capital Questionnaire; JES = Job Embeddedness Scale; M = Mean; SD = Standard Deviation

F = one-way ANOVA; t = independent samples t-test; η² = partial eta-squared (effect size for ANOVA); Cohen’s d = effect size for t-test

Effect sizes are interpreted as: small (η² ≈ 0.01, d ≈ 0.2), medium (η² ≈ 0.06, d ≈ 0.5), large (η² ≥ 0.14, d ≥ 0.8)

*: Statistically significant at *p* ≤ 0.05

Importantly, the number of patients per shift demonstrated a statistically significant negative association with JES (t = 3.404, *p* = 0.001, Cohen’s d = 0.48), suggesting that nurses with heavier patient loads reported lower levels of embeddedness. Monthly income was another significant factor for JES (F = 6.571, *p* = 0.002, η² = 0.030), with higher embeddedness scores reported by nurses with sufficient income. Additionally, the number of vacation days taken in the previous month was significantly associated with JES (F = 2.985, *p* = 0.012, η² = 0.013), with those taking no vacations or fewer days off generally reporting higher embeddedness. (Table 2)

In contrast, Psychological Capital (PCQ) showed fewer significant relationships. A notable finding was the strong association between PCQ and monthly income (F = 11.365, *p* < 0.001), indicating that nurses with sufficient income had significantly higher psychological capital. No statistically significant differences in PCQ were found based on gender, age, marital status, education level, working hours, patient load, or vacation days, although trends were observed in some categories (e.g., nurses aged < 20 reported the highest PCQ means, albeit not statistically significant). (Table 2)

Table 3 illustrates the distribution of nurses based on their levels of Psychological Capital, categorized into low, moderate, and high agreement levels. The findings reveal that the majority of nurses reported either moderate or high levels of Psychological Capital. Specifically, 44.3% of nurses (*n* = 191) fell within the moderate agreement category, while 43.4% (*n* = 187) reported high agreement with statements reflecting Psychological Capital. In contrast, only 12.3% (*n* = 53) of the nurses demonstrated low agreement, indicating lower levels of Psychological Capital.

**Table 3:** The levels of psychological capital among nurses

Psychological Capital	No	%	Mean Score
Low agreements	53	12.3	< 2.50
Moderate agreements	191	44.3	2.50 to < 3.75
High agreements	187	43.4	≥ 3.75

Low: Mean score < 2.50 Moderate: Mean score from 2.50 to < 3.75 High: Mean score ≥ 3.75

Table 4 presents the Pearson correlation coefficients between the four dimensions of Psychological Capital—self-efficacy, hope, resilience, and optimism—as well as their overall total, in relation to Job Embeddedness. The findings show strong, positive, and statistically significant correlations (*p* < 0.001) among all dimensions of Psychological Capital. For example, self-efficacy is highly correlated with hope (*r* = 0.551), resilience (*r* = 0.577), and optimism (*r* = 0.641). These strong intercorrelations suggest that the four dimensions are closely related and together form a cohesive construct. The overall Psychological Capital score, it is strongly correlated with its individual components: self-efficacy (*r* = 0.625), hope (*r* = 0.627), resilience (*r* = 0.610), and optimism (*r* = 0.620), all with statistical significance (*p* < 0.001). This confirms that each dimension significantly contributes to the overall measure of Psychological Capital.

**Table 4:** Correlation between study variables and their dimensions (*n* = 431)

		Self-efficacy	Hope	Resilience	Optimism	Overall Psychological Capital	Overall Job Embeddedness
Self-efficacy	r						
	p						
Hope	r	0.551*					
	p	< 0.001*					
Resilience	r	0.577*	0.588*				
	p	< 0.001*	< 0.001*				
Optimism	r	0.641*	0.652*	0.616*			
	p	< 0.001*	< 0.001*	< 0.001*			
Overall Psychological Capital	r	0.625*	0.627*	0.610*	0.620*		
	p	< 0.001*	< 0.001*	< 0.001*	< 0.001*		
Overall Job Embeddedness	r	0.272*	0.287*	0.339*	0.406*	0.356*	
	p	< 0.001*	< 0.001*	< 0.001*	< 0.001*	< 0.001*	
Mean ± SD		**3.73 ± 0.91**	**3.75 ± 0.84**	**3.73 ± 0.73**	**3.78 ± 0.67**	**3.75 ± 0.71**	**3.44 ± 0.74**

r: Pearson coefficient *: Statistically significant at *p* ≤ 0.05

In terms of their relationship with Job Embeddedness, all components of Psychological Capital show moderate, positive, and statistically significant correlations. Optimism has the strongest relationship with Job Embeddedness (*r* = 0.406, *p* < 0.001), followed by resilience (*r* = 0.339), hope (*r* = 0.287), and self-efficacy (*r* = 0.272). The total Psychological Capital score also shows a significant positive correlation with Job Embeddedness (*r* = 0.356, *p* < 0.001), indicating that as Psychological Capital increases, so does the level of Job Embeddedness. The mean scores for each Psychological Capital dimension and Job Embeddedness also reflect generally high levels among the sample, with Psychological Capital dimensions averaging between 3.73 and 3.78, and Job Embeddedness at 3.44 (Table 4).

The results of the multiple linear regression analysis, as presented in Table 5, revealed that the overall model was statistically significant (F(7, 423) = 14.13, *p* < 0.001), accounting for approximately 19.8% of the variance in nurses’ job embeddedness (R² = 0.198, Adjusted R² = 0.195). Among the variables examined, psychological capital showed the strongest and most statistically significant positive linking with job embeddedness (B = 0.317, t = 6.084, *p* < 0.001, 95% CI: [0.214, 0.419]). This finding suggests that nurses with higher levels of psychological capital—comprising hope, resilience, optimism, and self-efficacy—tend to report a greater sense of attachment to their job and organization.

**Table 5:** Linear regression on the effect of psychological capital on job embeddedness (*n* = 431)

Predictors	B	SE	t	*p*	95% CI	
					LL	UL
Marital status (Married)	-0.115	0.063	-1.818	0.070	-0.240	0.009
Level of edsucation	-0.027	0.057	-0.468	0.640	-0.139	0.085
Number of working hours /week	-0.041	0.038	-1.071	0.285	-0.117	0.034
Number of patients in your shift	-0.269	0.076	-3.529*	< 0.001*	-0.419	-0.119
Income	-0.048	0.051	-0.936	0.350	-0.149	0.053
Number of vacation last month	-0.023	0.023	-1.037	0.301	-0.068	0.021
Psychological Capital	0.317	0.052	6.084*	< 0.001*	0.214	0.419

Model Summary: R² = 0.198, Adjusted R² = 0.195, F (7, 423) = 14.13, *p* < 0.001

R^2^: Coefficient of determination, B: Unstandardized Coefficients, se: standard error, t: t-test of significance, LL: Lower limit, UL: Upper Limit, CI: confidence interval, #: reference for dummy coded variable, *: Statistically significant at *p* ≤ 0.05

In contrast, the number of patients per shift demonstrated a statistically significant negative association with job embeddedness (B = -0.269, t = -3.529, *p* < 0.001, 95% CI: [-0.419, -0.119]), indicating that heavier patient loads are linked to lower levels of organizational attachment, possibly due to increased job strain and reduced engagement. Other demographic and work-related characteristics—including marital status, level of education, number of working hours per week, monthly income, and number of vacation days—were not significantly linked to with job embeddedness (*p* > 0.05). While marital status approached statistical significance (*p* = 0.070), it did not meet the threshold for inclusion as a meaningful correlate in this model.

## Discussion

Nursing care has significantly benefited from PsyCap, which includes self-efficacy, hope, optimism, and resilience [17]. This study aimed to explore the relationship between PsyCap and JE among nurses in Egyptian governmental hospitals. The findings indicated a significant positive association between nurses’ PsyCap and their sense of job embeddedness, suggesting that nurses with higher levels of resilience, optimism, hope, and self-efficacy feel more anchored to their roles and organizations.

The examination of demographic factors in relation to Psychological Capital and Job Embeddedness provides valuable insights into their interactions within the nursing workforce. Interestingly, gender did not show significant differences in either PsyCap or JE, indicating that both male and female nurses exhibit comparable levels of psychological resources and job attachment. This observation aligns with existing research that indicates PsyCap is largely unaffected by gender, highlighting the necessity for healthcare organizations to implement universal strategies that cultivate these attributes across the workforce [18]. By promoting collective development rather than focusing on gender-specific interventions, healthcare institutions can enhance overall employee engagement and retention.

Age also did not significantly influence either PsyCap or JE, indicating that nurses of varying ages maintain similar psychological capital and job attachment. This denotes that the cultivation of psychological resources is not limited to younger or less experienced nurses but can be nurtured throughout all stages of a nursing career. Research has shown that both younger and older nurses contribute unique strengths to the workplace, with younger nurses often displaying adaptability while older nurses bring invaluable experience [19]. Therefore, initiatives aimed at enhancing PsyCap should be inclusive, leveraging the diverse strengths present in the entire nursing workforce.

Marital status emerged as a significant factor affecting job embeddedness, with single nurses demonstrating higher levels of attachment to their jobs compared to married or divorced/widowed nurses. This may be due to fewer personal commitments allowing single nurses to engage more deeply with their work environment. Studies indicate that personal circumstances significantly influence job satisfaction and attachment, emphasizing the need for healthcare organizations to consider these dynamics in their retention strategies. Tailoring support and resources to address the specific needs of nurses based on their marital status can foster a more committed and engaged workforce [20, 21].

The analysis also highlighted the influence of income and workload on both Psychological Capital and Job Embeddedness. A higher income positively correlated with both psychological capital and job embeddedness, underscoring the importance of financial stability in enhancing nurses’ psychological resources. Research suggests that financial stress can negatively impact overall job satisfaction and well-being [18]. Additionally, the number of patients per shift was found to negatively affect job embeddedness, indicating that increased workloads can lead to burnout and diminish emotional connections to the job. This aligns with findings that excessive workload can reduce job satisfaction and retention [22].

Moreover, The inverse relationship between workload and job embeddedness may be interpreted through the lens of the Job Demands-Resources (JD-R) model. According to this framework, excessive job demands—such as high patient loads—can deplete employees’ physical and emotional resources, leading to strain, disengagement, and ultimately, a weakened attachment to the organization. In this context, workload functions as a hindrance demand that undermines nurses’ capacity to invest in their roles and develop the relational and emotional ties that characterize job embeddedness [23].

The distribution of Psychological Capital levels among nurses indicates a significant prevalence of moderate to high agreements, suggesting that many nurses possess positive psychological resources. With nearly half of the respondents showing moderate levels of agreement, it is evident that nurses recognize the importance of hope, resilience, optimism, and self-efficacy in their roles. This is consistent with existing literature that highlights the benefits of Psychological Capital in enhancing workplace engagement and mitigating burnout, especially in high-stress environments like healthcare [24, 25]. By fostering these psychological attributes, healthcare organizations can enhance job satisfaction and performance among nurses, ultimately improving patient care.

However, the presence of a notable percentage of nurses reporting low agreements raises concerns. This group may encounter barriers that inhibit the development of their Psychological Capital, potentially impacting their job satisfaction and well-being. Addressing the factors that contribute to low Psychological Capital is essential, as research indicates that diminished psychological resources can lead to increased turnover intentions and reduced organizational commitment. Therefore, targeted interventions, such as training programs focused on resilience and optimism, are vital for cultivating a more engaged and stable nursing workforce [7].

The correlation analysis among the dimensions of Psychological Capital—self-efficacy, hope, resilience, and optimism—reveals strong interrelationships that highlight the cohesive nature of these constructs. The high correlation coefficients suggest that as one dimension increases, others tend to rise as well, indicating that these psychological resources support and strengthen each other. For instance, self-efficacy is closely linked to hope, denoting that nurses who feel confident in their abilities are more likely to maintain a hopeful outlook regarding their futures in the profession. This synergy aligns with the HERO framework, which posits that these positive psychological states are vital for enhancing employee performance and well-being in challenging environments like healthcare [3].

Furthermore, the significant correlation between overall Psychological Capital and overall Job Embeddedness underscores the critical role psychological resources play in fostering job attachment. The positive relationship indicates that nurses with higher levels of Psychological Capital are more likely to feel connected to their organizations and committed to their roles. This finding is consistent with existing literature that emphasizes the importance of Psychological Capital in reducing turnover intentions and enhancing organizational commitment [26, 27]. By investing in the development of these psychological resources, healthcare organizations can improve individual nurse outcomes and promote greater job satisfaction and retention within the nursing workforce.

The linear regression analysis demonstrates a significant positive effect of Psychological Capital on Job Embeddedness, indicating that higher levels of psychological resources among nurses are associated with stronger job attachment. This finding is particularly noteworthy, as it emphasizes the role of Psychological Capital as a key predictor of Job Embeddedness. The positive coefficient suggests that for every unit increase in Psychological Capital, nurses’ sense of job attachment increases significantly. This supports existing literature that underscores the significance of psychological resources in enhancing employee engagement and retention, especially in high-stress professions like nursing [28].

Conversely, the analysis reveals that several demographic and workload-related factors, such as marital status, education level, and income, do not significantly assosicted with Job Embeddedness. Notably, the number of patients per shift emerged as a significant negative predictor, highlighting the adverse impact of high patient loads on nurses’ emotional connections to their jobs. This aligns with research indicating that excessive workloads are linked to increased stress and burnout, which can undermine job satisfaction and commitment [29, 30]. Thus, while Psychological Capital is a vital resource for fostering job embeddedness, addressing workload challenges is equally crucial for enhancing overall job satisfaction and retention in the healthcare setting [31, 33, 34].

### Study limitations and recommendations for future research

Firstly, it used a cross-sectional survey methodology and a convenience sample approach, which limits its ability to establish causal relationships and apply its findings more broadly. Using a self-administered questionnaire could have introduced bias in the participant responses. Future studies should employ longitudinal or experimental designs to explore causal pathways between psychological capital and job embeddedness. Intervention studies aimed at enhancing psychological capital could provide valuable insights into whether improvements in PsyCap lead to sustained increases in job embeddedness over time.

A more diverse sample is crucial to ensure a more comprehensive understanding. Finally, the response rate was high (95.5%), this may reflect selection bias. Though participants were explicitly assured of voluntary participation and confidentiality, the hierarchical nature of healthcare settings may still introduce a risk of perceived coercion, especially if staff felt compelled to participate due to institutional culture or peer pressure. Future studies should consider anonymous online or self-administered digital surveys to mitigate this risk further.

Moreover, the study was conducted in only two hospitals within one Egyptian governorate, limiting its representativeness. Consequently, findings may not generalize to other regions, private healthcare settings, or international contexts. Broader multi-site and longitudinal research is recommended to enhance external validity and to explore causality more robustly.

This study did not assess potential mediators or moderators of the PsyCap–JE relationship, such as burnout, job satisfaction, or workload. Future research should incorporate these variables to better understand the mechanisms and conditional factors influencing this relationship.

## Conclusions

The findings highlight the crucial role of psychological capital in fostering job embeddedness among nurses in Egyptian public hospitals. Psychological capital, comprising hope, resilience, self-efficacy, and optimism, appears to be a key factor in strengthening nurses’ connections to their workplace connections. This suggests that healthcare organizations could benefit from implementing strategies to enhance psychological resources among their nursing staff. The negative association between increased patient load and job embeddedness underscores the importance of addressing workload issues in health care settings. High patient-to-nurse ratios not only compromise quality of care but also erode nurses’ sense of attachment to their organization.

### Implications for nursing practice and health policy

Healthcare organizations can play a pivotal role in enhancing nurses’ psychological capital (PsyCap) by implementing structured training programs that focus on developing resilience, optimism, and self-efficacy. Investing in these psychological resources may support nurses in coping with workplace stressors, contributing to higher levels of job embeddedness and, ultimately, improved retention and patient care outcomes. Furthermore, initiatives aimed at fostering social capital—such as improving team communication and promoting supportive work relationships—may also contribute to a more engaging and resilient workforce [32, 35, 36].

In addition to psychological interventions, structural factors such as workload must be addressed. Given the significant negative association between patient load and job embeddedness observed in this study, it is essential that healthcare administrators explore practical strategies to reduce excessive workloads. Drawing on the Job Demands-Resources (JD-R) model, high patient loads act as a job demand that can erode employees’ psychological resources and weaken their organizational attachment.

organizations could implement a workload reduction intervention (e.g., improving nurse-to-patient ratios or redistributing non-clinical tasks) and examine whether such changes enhance job embeddedness. This effect could be mediated by improvements in psychological capital or perceived organizational support. Such evidence would provide stronger causal insight and inform more effective, evidence-based staffing and retention policies.

## Data Availability

Data will be available on reasonable request from the corresponding author.
